# S02-4: How does the Hungarian Active Lifestyle Strategy address social inclusion?

**DOI:** 10.1093/eurpub/ckae114.205

**Published:** 2024-09-26

**Authors:** Gyöngyvér Lacza, Réka Veress

**Affiliations:** University of Sport Sciences; National School, University and Leisure Sport Federation

## Abstract

**Purpose:**

The purpose of Sarolta MONSPART Active Lifestyle Strategy is to raise proportion of physically active people with a healthy lifestyle in Hungary. There is special focus on the engagement of senior citizens and people with disadvantaged background.

**Project description:**

The Strategy is the first ever national policy document dedicated to the field of overall promotion of health-enhancing physical activities.

**Methods:**

The development of the Strategy had been initiated by the State Secretary for Active Lifestyle. The University of Sport Sciences had been invited for the coordination of the Strategy’ development, which is based on outcomes of international policy document analysis and national stakeholder consultations. The Strategy identifies ten priority target areas and eight horizontal priorities. “Active seniors” and “Activation of socially disadvantaged groups”, which includes people with disabilities and people with unfavourable socio-economic background are also priority areas.

The implementation of the Strategy is planned to happen on the base of the proposed actions in all priority target areas. Respective governmental and non-governmental organisations have been involved into the drafting of the Strategy. Based on their proposals for implementing actions, an action plan has been drafted. Respectively these organisations are also important actors during the delivery of the proposed initiatives.

The evaluation of the implementation of the Strategy will be based on the proposed indicators, that are part of the Strategy in relation to each proposed action, also reflecting on the number of successfully implemented actions and number of participants.

Dissemination of the strategy has already started during the drafting phase, by the involvement of ca. 50 organisations as consultative partners. After the official approval of the Strategy, communication will be ensured by the State Secretariat for Active Lifestyle and the implementing partners.

**Conclusions:**

The State Secretariat for Active Lifestyle will monitor the implementation of the action plan and collect the well-functioning methods and practices. It is expected that a relevant network of professional organisations will be created in all priority target fields and those networks will be able to provide data and feedback on the proposed actions.

